# The thrombopoietin receptor S264F mutant drives MPN with marrow fibrosis through a dual mechanism

**DOI:** 10.1002/hem3.70437

**Published:** 2026-10-07

**Authors:** Charlotte Lees, Emile Brandt, Emmanuel Dutranoix, Gabriel Levy, Bogdan I. Iorga, Hosuk Ryou, Jens Rittscher, Daniel Royston, Stefan N. Constantinescu, Adam J. Mead

**Affiliations:** ^1^ MRC Molecular Haematology Unit, MRC Weatherall Institute of Molecular Medicine, Radcliffe Department of Medicine and National Institute of Health Research (NIHR), Oxford Biomedical Research Centre University of Oxford Oxford United Kingdom; ^2^ de Duve Institute Université Catholique de Louvain Brussels Belgium; ^3^ Ludwig Institute for Cancer Research Brussels Belgium; ^4^ Institut de Chimie des Substances Naturelles Université Paris‐Saclay, CNRS Gif‐sur‐Yvette France; ^5^ Nuffield Division of Clinical Laboratory Sciences, Radcliffe Department of Medicine University of Oxford Oxford United Kingdom; ^6^ Institute of Biomedical Engineering (IBME), Department of Engineering Science University of Oxford Oxford United Kingdom; ^7^ Ludwig Institute for Cancer Research, Nuffield Department of Medicine Oxford University Oxford United Kingdom; ^8^ Big Data Institute University of Oxford, Li Ka Shing Centre for Health Information and Discovery Oxford United Kingdom; ^9^ Oxford NIHR Biomedical Research Centre Oxford University Hospitals Trust Oxford United Kingdom; ^10^ Oxford University Hospitals NHS Foundation Trust Oxford United Kingdom; ^11^ Walloon Excellence in Life Sciences and Biotechnology Department Walloon Excellence Research Institute Wavre Belgium

The thrombopoietin (TPO)/TPO receptor (TPOR/MPL) axis is fundamental for megakaryocyte (MK) differentiation, platelet production, and maintenance of hematopoietic stem cells. TPO is produced in the kidney, liver, and bone marrow and binds the extracellular domain (ECD) of TPOR on the cell surface, leading to receptor dimerization and activation of the JAK‐STAT pathway.^1^ Upon binding its receptor on MKs and platelets, TPO is internalized and degraded, regulating its level via a negative feedback mechanism. Hence, in normal human hematopoiesis, TPO levels correlate inversely with platelet count.^2^

TPOR is encoded by the *MPL* gene on chromosome 1. Activating germline mutations of TPOR have been implicated in hereditary thrombocytosis.^1^ Somatic TPOR mutations are phenotypic driver mutations in myeloproliferative neoplasms (MPN), occurring in 5%–7% essential thrombocythemia (ET) cases, classically at positions 515 and 505 in exon 10, which covers the transmembrane domain (TMD),^3^ leading to constitutive JAK‐STAT activation^4^, ^5^ and an MPN phenotype in mouse models with an increase in MK and platelet numbers.^6^, ^7^

Conversely, homozygous knockout of *MPL* in mice causes significant thrombocytopenia and high TPO levels due to a lack of receptor‐mediated clearance.^1^ In humans, inherited inactivating TPOR mutations are implicated in congenital amegakaryocytic thrombocytopenia (CAMT), an autosomal recessive disease characterized by thrombocytopenia and progression to trilineage aplasia; these patients also exhibit high TPO levels. Most mutations in CAMT are missense mutations, predominantly affecting TPO‐binding residues 102 and 104, causing absent cell surface expression or inability to bind TPO, respectively.^8^ Intriguingly, reduced surface TPOR due to germline mutations can paradoxically result in thrombocytosis.^1^ For example, the CAMT mutation *MPL* R102P exerts the opposite phenotype of thrombocytosis when heterozygous. It is thought that the partial trafficking defect leads to insufficient clearance of TPO, which drives thrombocytosis via the remaining wild‐type (WT) receptor.^9^

We present a somatic *MPL* mutation not previously reported in MPN, identified in a 51‐year‐old white British male, who presented clinically well but with an incidental thrombocytosis of 840 × 10^9^/L. Other blood parameters were normal (Supporting Information S1: Figure S1). Blood film examination confirmed thrombocytosis but was otherwise unremarkable. Bone marrow trephine demonstrated MPN features with grade MF1–MF2 fibrosis but no excess of blasts. Using Continuous Indexing of Fibrosis (CIF) analysis,^10^ this trephine lies in the prefibrotic primary myelofibrosis (pre‐PMF) region of PCA space (Figure 1A,B). He had a normal karyotype. A 67‐gene next‐generation sequencing panel did not identify canonical driver mutations but detected the *MPL* c.791C>T p.(S264F) missense mutation at a variant allele frequency (VAF) of 44%. This variant has been reported before in hematological malignancy, but only in pediatric acute myeloid leukemia with dysplasia.^11^ Analysis of T cells demonstrated a much lower VAF (2.4%), supporting somatic origin. He commenced pegylated interferon, achieving a sustained complete hematological response after 13 months of 45 μg every other week. There have been serial reductions in VAF to 32% after 13 months and 24% after 23 months.

**Figure 1:**
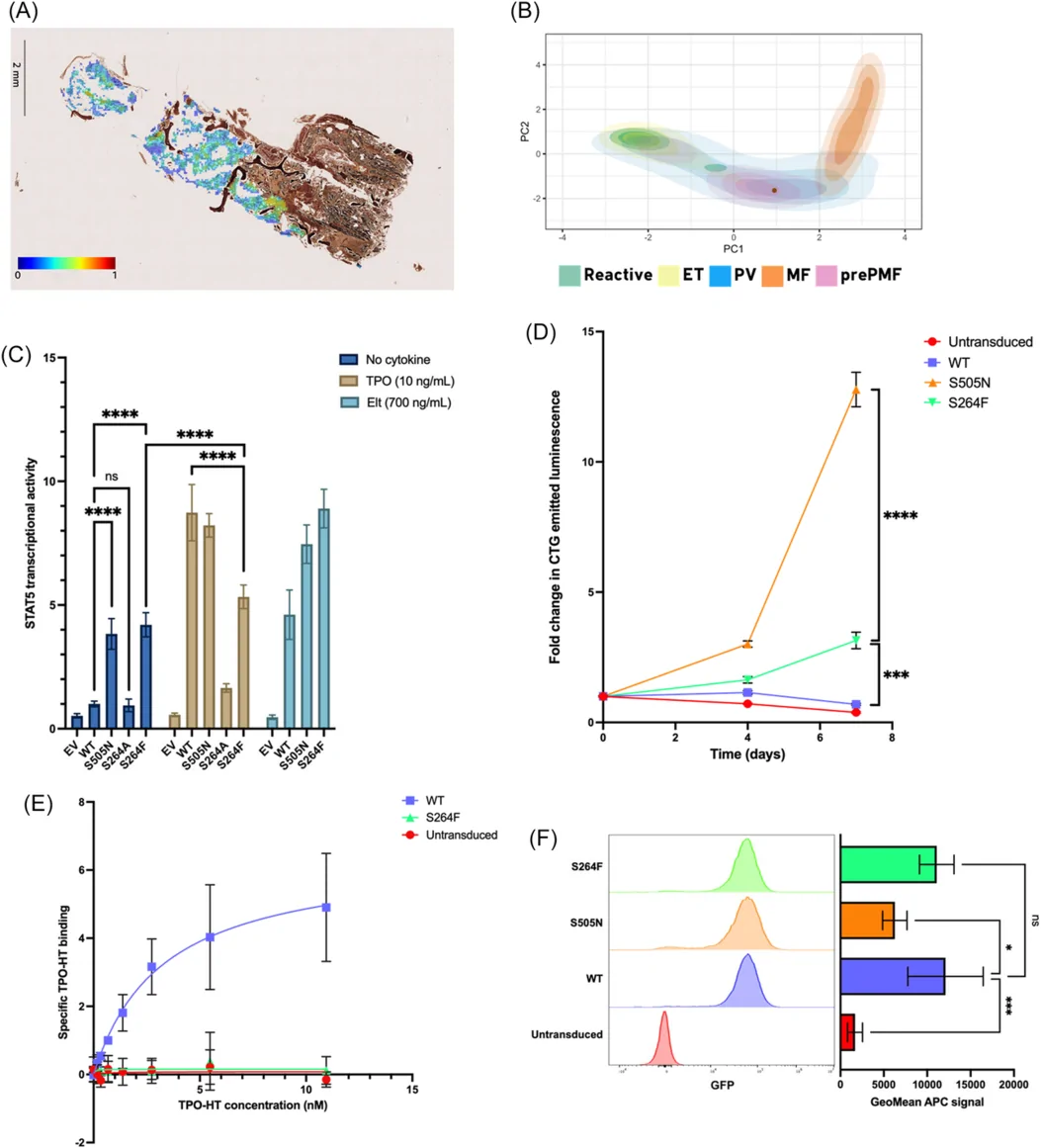
**S264F induces constitutive TPOR signaling and impairs TPO binding in a myeloproliferative neoplasm context**. **(A)** Fibrosis severity mapping in bone marrow trephine using continuous indexing of fibrosis (CIF), overlaid onto an H&E‐stained section. Fibrosis severity was normalized to a scale from 0 (no fibrosis) to 1 (severe fibrosis). **(B)** Principal component analysis (PCA) of fibrosis phenotype showing the position of the patient's trephine (red dot) within the myeloproliferative neoplasm (MPN) landscape. The analysis integrates average fibrosis severity and spatial heterogeneity, and is compared to a reference cohort of MPN samples previously characterized at Oxford University Hospitals NHS Trust. **(C)** JAK‐STAT pathway activation in HEK293T cells transiently transfected with hemagglutinin (HA)‐tagged TPOR variants together with JAK2, STAT5, and a firefly luciferase reporter plasmid (pSPI‐LUC) driven by STAT5‐responsive elements. Cells were stimulated with TPO, eltrombopag (Elt), or left unstimulated. Luciferase activity was normalized to Renilla luciferase (pRL‐TK) as an internal control. **(D)** Proliferation of Ba/F3 cells expressing TPOR variants was assessed over 7 days using a CellTiter‐Glo luminescent cell viability assay. Data represent mean ± SD of triplicate cultures. **(E)** Binding of C‐terminal HaloTag‐fused TPO (TPO‐HT) to Ba/F3 cells expressing TPOR variants. Cells were incubated with increasing concentrations of TPO‐HT labeled with Alexa Fluor 660. Binding was quantified by flow cytometry. Specific binding was determined by competition with excess untagged TPO and calculated as the difference in fluorescence signal between competed and non‐competed conditions in GFP‐positive cells. **(F)** Cell surface localization of TPOR in Ba/F3 cells was assessed by flow cytometry using an APC‐conjugated anti‐HA antibody after sorting for comparable GFP expression levels. Unless otherwise indicated, data represent mean ± SD of 3–4 independent experiments. Statistical analysis was performed using one‐way or two‐way ANOVA with Tukey's multiple comparisons test. ns, not significant; *P < 0.05; ***P < 0.001; ****P < 0.0001. ANOVA, analysis of variance; TPOR, thrombopoietin receptor.

Plasma TPO level was 81.3 pg/mL (Human Thrombopoietin Quantikine ELISA Kit; R&D Systems). Plasma TPO levels in healthy individuals are usually undetectable (79%), with a mean detectable level of 91.8 pg/mL for this assay (range: not detected‐196 pg/mL; R&D Systems). With the thrombocytosis exhibited in this case, 81.3 pg/mL is inappropriately high.

The 3.4 Å resolution cryo‐electron microscopy structure of the extracellular human TPO/TPOR complex was recently reported.^12^ The high‐ and low‐affinity interfaces, referred to as regions binding TPO sites 1 and 2, identified serine 264 as a TPO‐binding residue shared in both regions. S264 is a residue of the second fibronectin type 3‐like domain (D2) of TPOR and is part of a loop facing TPO when binding its receptor.

Functional experiments using dual‐luciferase STAT5 reporter gene assays in transiently transfected HEK293T cells demonstrated that the TPOR S264F mutant, unlike S264A, induces constitutive activation of the JAK‐STAT pathway. This was evidenced by increased STAT5 transcriptional activity under unstimulated conditions compared to WT TPOR, at levels comparable to the canonical activating mutant S505N (Figure 1C). Upon TPO stimulation, however, S264F showed only a very modest additional increase in STAT5 activity (Figure 1C). A similar activation pattern was observed in a STAT3 reporter assay (Supporting Information S1: Figure S2). Protein expression levels of the WT and different TPOR mutants were confirmed by western blot (Supporting Information S1: Figure S3). A 7‐day proliferation assay using stably transduced Ba/F3 cells showed that S264F supports IL‐3‐independent growth, albeit to a lesser extent than S505N (Figure 1D).

To assess ligand binding, we performed a flow cytometry‐based TPO‐HaloTag (TPO‐HT)/TPOR binding assay in stably transduced Ba/F3 cells using Alexa Fluor 660 (AF660)‐labeled TPO‐HT. Expression of TPO‐HT produced in HEK293T cell supernatant was confirmed and semi‐quantitatively assessed by western blot (Supporting Information S1: Figure S4). Its biological activity was validated using a STAT5 luciferase assay (Supporting Information S1: Figure S5). Specific binding was further confirmed by competition with an excess of untagged TPO, which reduced the AF660 signal only in WT TPOR‐expressing cells (Supporting Information S1: Figure S6). WT TPOR‐expressing cells displayed saturable specific binding with an apparent Kd of ~3 nM. In contrast, cells expressing TPOR S264F and untransduced control cells showed no detectable specific binding or saturation across the tested concentration range (Figure 1E). However, we cannot exclude very low‐affinity binding that is not detected by our assay.

Western blot and flow cytometry analyses confirmed comparable total protein expression levels and cell surface localization of WT and S264F TPOR, indicating that the mutation does not impair protein expression or trafficking (Figure 1F and Supporting Information S1: Figure S7). In WT TPOR‐expressing cells, increasing concentrations of TPO‐HT were associated with a decrease in surface TPOR levels, consistent with ligand‐induced receptor internalization. In contrast, TPOR S264F surface levels remained stable across all conditions, consistent with impaired ligand binding (Supporting Information S1: Figure S8).

Notably, consistent with the STAT5 luciferase assay, S264F retained a detectable response in a TPO dose‐response assay compared to WT (Supporting Information S1: Figure S9). This suggests residual low‐level ligand binding below the detection limit of the binding assay, yet sufficient to trigger downstream signaling at high concentrations.

Eltrombopag, a small‐molecule TPOR agonist, interacts with juxtamembrane extracellular residues to induce an alpha‐helical conformation in a region preceding H499, and thereby promotes receptor dimerization and activation similar to S505N.^5^ We conducted a dose‐response assay with eltrombopag and observed increased sensitivity to the drug, reflected by a lower EC50 (51.4 ng/mL−91.0 nM) compared to WT TPOR (327 ng/mL−579 nM) (Figure 2A). This finding is compatible with a primed predimerized state induced by S264F. In line with this, TPOR S264F induced an increased response to eltrombopag in the STAT5 reporter assay (Figure 1C).

**Figure 2:**
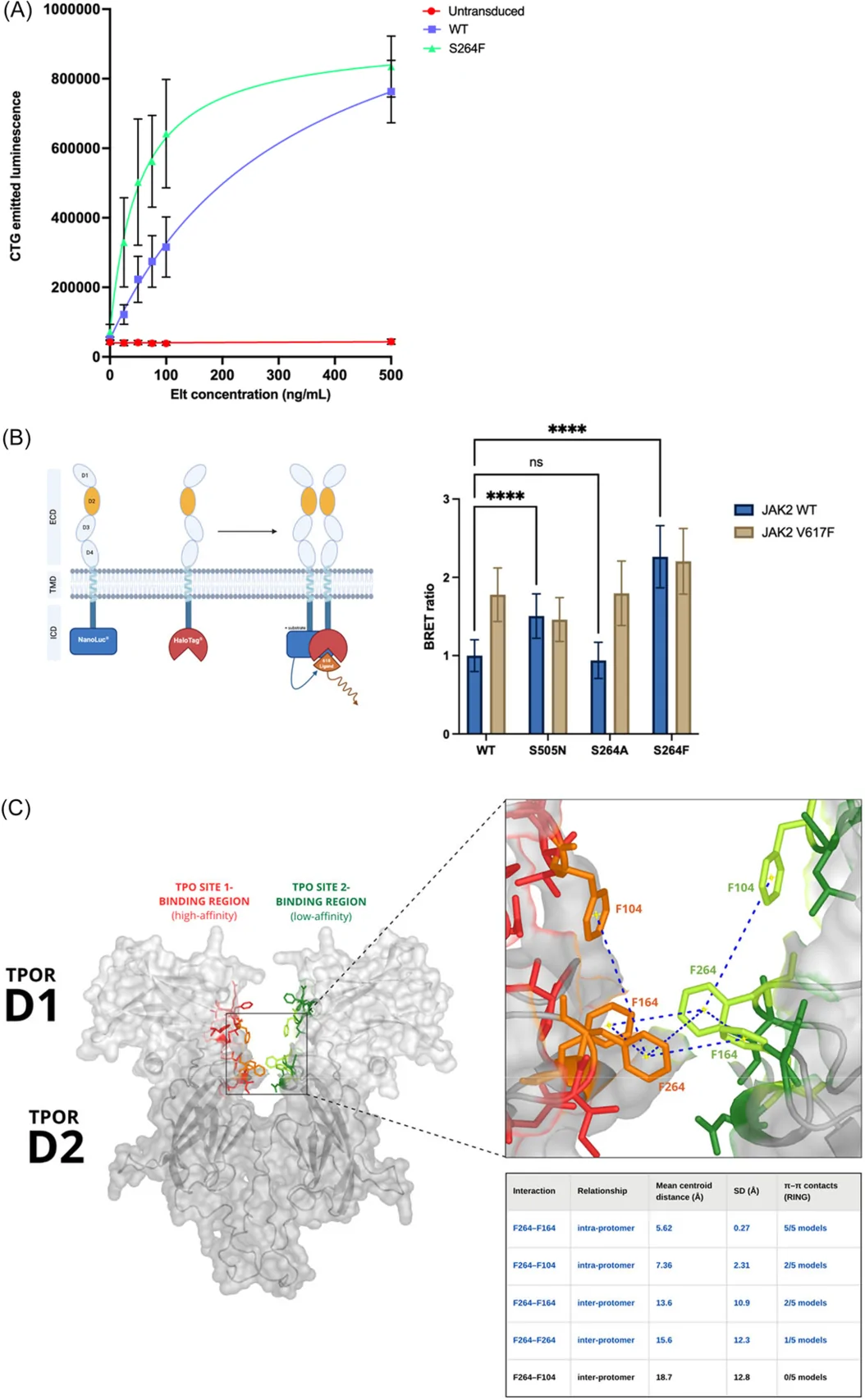
**Mechanistic insights into TPOR S264F function and structure. (A)** Eltrombopag (Elt) dose‐response in Ba/F3 cells expressing TPOR variants. Cell proliferation was measured after 3 days using a CellTiter‐Glo luminescent cell viability assay in the presence of increasing concentrations of Elt. EC50 values were calculated using a three‐parameter non‐linear regression model. **(B)** Ligand‐independent TPOR dimerization was assessed by NanoLuc‐based bioluminescence resonance energy transfer (NanoBRET) in HEK293T cells between TPOR constructs fused to NanoLuc donor or HaloTag acceptor at the cytoplasmic domain. Dimerization was quantified by measuring the BRET ratio. JAK2 V617F‐induced TPOR dimerization was used as a positive control. **(C)** AlphaFold3 structural modeling of two S264F TPOR protomers encompassing extracellular domains D1 and D2. The model highlights a potential aromatic interaction network within TPOR regions located at the interfaces with TPO binding site 1 (high‐affinity) and site 2 (low‐affinity), shown in red and green, respectively. This interaction network may contribute to constitutive receptor dimerization and enhanced Elt responsiveness of the TPOR S264F mutant. Residues F104, F164, and F264 from the high‐affinity interface are shown in orange, while the corresponding residues from the low‐affinity interface are shown in light green. Aromatic ring centroids are represented as yellow stars, and centroid‐to‐centroid distances are depicted as blue dashed lines when compatible with *π*–*π* interactions and/or supported by RING analysis. Structural analyses were performed using five independent AlphaFold3 predictions. Unless otherwise indicated, data represent mean ± SD of three independent experiments. Statistical analysis was performed using two‐way ANOVA with Tukey's multiple comparisons test. ns, not significant; ****P < 0.0001. ANOVA, analysis of variance.

To determine whether S264F promotes TPOR dimerization, we performed a NanoBRET assay using TPOR constructs fused to a C‐terminal NanoLuc donor and HaloTag acceptor. Our data show that the S264F mutation resulted in enhanced dimerization of TPOR intracellular domains, as evidenced by a higher BRET ratio than WT TPOR (Figure 2B). TPOR constructs showed comparable total protein and surface expression levels (Supporting Information S1: Figure S10 and S11). The JAK2 V617F mutant was included as a positive control for TPOR dimerization (Figure 2B).

Computational modeling using AlphaFold3^13^ suggested a local aromatic interaction network within the TPOR ECD involving residues F104, F164, and the mutant F264 (Figure 2C). We measured centroid‐to‐centroid distances between aromatic rings across five independent AlphaFold3 models. Distances compatible with *π*–*π* interactions were consistently observed between intra‐protomer F264 and F164 (mean 5.62 Å), whereas distances involving F104 or inter‐protomer F164/F264 were more variable and often exceeded the expected range for stable interactions. To complement this analysis, we used RING^14^ to identify noncovalent contacts. Intraprotomer *π*–*π* interactions between F264 and F164 were detected across all models, while interactions involving F104 or inter‐protomer F164/F264 were less frequently observed. Accordingly, introduction of the F164A mutation in the S264F background abolished the constitutive activation of the JAK‐STAT pathway induced by the S264F single mutant as detected by the STAT5 luciferase reporter assay (Supporting Information S1: Figure S12), without reduced cell surface localization between receptor variants (Supporting Information S1: Figure S13). In contrast, the F104A mutation did not suppress S264F‐mediated TPOR activation, further supporting our model. Collectively, these data support a model in which the introduction of an aromatic residue at position 264 favors *π*–*π* interactions, contributing to local structural stabilization within the ECD. These constraints may influence the transmembrane alpha‐helical dimer interface toward a conformation permissive for TPO‐independent activation, enabling spontaneous dimerization and downstream signaling.

In conclusion, our findings demonstrate the functional relevance of the TPOR S264F mutation in the patient's disease, acting via a dual mechanism. First, S264F impairs TPO binding and receptor‐mediated clearance, resulting in relatively high plasma TPO levels despite thrombocytosis, which may contribute to paradoxical ligand‐driven thrombopoiesis through residual WT receptor. Second, the mutation appears to induce constitutive TPOR signaling, probably through an extracellular‐domain conformational change that propagates to the transmembrane dimer interface.

Many patients with MPN lack clear pathogenic variants and are therefore classified as triple‐negative. Here, we demonstrate that TPOR S264F is likely the driver of pre‐PMF in this patient. Investigating non‐canonical TPOR mutations, particularly those affecting the ECD, is essential as they reveal critical extracellular regions that prevent autonomous receptor activation and could identify novel therapeutic targets.

## AUTHOR CONTRIBUTIONS

**Charlotte Lees**: Writing—original draft; investigation; writing—review and editing; visualization; formal analysis; methodology; project administration. **Emile Brandt**: Writing—original draft; writing—review and editing; investigation; methodology; formal analysis; visualization; validation. **Emmanuel Dutranoix**: Investigation; writing—review and editing; visualization; methodology. **Gabriel Levy**: Methodology; investigation; writing—review and editing. **Bogdan I. Iorga**: Methodology; writing—review and editing; visualization; investigation. **Hosuk Ryou**: Investigation; methodology; writing—review and editing; formal analysis; visualization; software. **Jens Rittscher**: Methodology; investigation. **Daniel Royston**: investigation; methodology; writing—review and editing; formal analysis; visualization. **Stefan N. Constantinescu**: Conceptualization; supervision; writing—review and editing; resources. **Adam J. Mead**: Conceptualization; supervision; writing—review and editing; resources.

## CONFLICT OF INTEREST STATEMENT

Stefan N. Constantinescu is co‐founder of MyeloPro Diagnostics and Research GmbH, Vienna, Austria. Jens Rittscher is a co‐founder of Ground Truth Labs. Other authors declare no conflict of interests.

## ETHICS STATEMENT

The patient gave written consent to the INForMeD Study (REC reference 16/LO/1376).

## FUNDING

Funding to SNC is acknowledged from the Ludwig Institute for Cancer Research, Fondation contre le Cancer Belgium, Fondation Salus Sanguinis, Fondation “Les Avions de Sébastien,” Actions de Recherche Concertées (ARC) 16/21‐073, Fonds de la Recherche Scientifique—FNRS (grants PDR T.0043.21 and U.G008.22), and Wel Research Institute WELBIO grant F 44/8/5—MCF/UIG—10955. EB holds a PhD fellowship from the Fondation “Les Avions de Sébastien.” CL holds a clinical research training fellowship from the Medical Research Council (MRC), UK. This work was supported by the National Institute for Health Research (NIHR) Oxford Biomedical Research Centre. Computation used the Oxford Biomedical Research Computing (BMRC) facility, a joint development between the Wellcome Centre for Human Genetics and the Big Data Institute, supported by Health Data Research UK and the NIHR Oxford Biomedical Research Centre. J.R. is an adjunct professor of the Ludwig Oxford Branch. This work was supported by a CRUK Senior Cancer Research Fellowship to AJM. (Grant number C42639/A26988) and a CRUK Discovery Programme Award to AJM. (Grant number DRCNPG‐Nov24/100003). The authors would like to acknowledge the CRUK Oxford Centre (CTRQQR‐2021\100002) and the National Institute for Health Research (NIHR) Oxford Biomedical Research Centre (BRC). The views expressed are those of the authors and not necessarily those of the National Health Service (NHS), the NIH, the NIHR, or the Department of Health and Social Care.

## Supporting information


**Supplementary Figure S1. Peripheral blood counts at diagnosis**; **Supplementary Figure S2. STAT3 reporter gene assay in HEK293T cells**. HEK293T cells were transiently transfected with empty vector (EV) or hemagglutinin (HA)‐tagged TPOR constructs, together with JAK2 and STAT3, and a STAT3‐responsive luciferase reporter (pGL4.47). Cells were stimulated with TPO, eltrombopag (Elt), or left unstimulated. Luciferase activity was normalized to Renilla luciferase (pRL‐TK) as an internal control. Data represent mean ± SD of three independent experiments performed in triplicate. Statistical analysis was performed using two‐way ANOVA with Tukey's multiple comparisons test. ns, not significant; **P < 0.01; ****P < 0.0001; **Supplementary Figure S3. TPOR protein expression in HEK293T cells**. Western blot analysis of TPOR expression in HEK293T cells transiently transfected with the indicated constructs; **Supplementary Figure S4. Estimation of TPO‐HaloTag concentration**. Western blot analysis of serial dilutions of TPO‐HaloTag (TPO‐HT)‐containing supernatant (top) alongside a HaloTag standard protein at known concentrations (bottom). Band intensities were compared within the linear detection range of the assay to estimate the concentration of TPO‐HT in the original supernatant; **Supplementary Figure S5. STAT5 reporter gene assay in HEK293T cells**. JAK‐STAT pathway activation in HEK293T cells transiently transfected with HA‐tagged TPOR variants together with JAK2, STAT5, and a firefly luciferase reporter plasmid (pSPI‐LUC) driven by STAT5‐responsive elements. Cells were stimulated with TPO‐HT or left unstimulated. Luciferase activity was normalized to Renilla luciferase (pRL‐TK) as an internal control. Data represent mean ± SD of three independent transfection experiments. Statistical analysis was performed using two‐way ANOVA with Tukey's multiple comparisons test. ****P < 0.0001; **Supplementary Figure S6. Flow cytometry‐based competitive ligand‐binding assay**. Ba/F3 cells expressing TPOR variants were incubated with Alexa Fluor 660‐labeled TPO‐HT in the presence or absence of excess untagged TPO. Specific binding was defined as the difference in geometric mean fluorescence intensity between conditions. Data represent geometric mean ± SD of three independent experiments. Statistical analysis was performed using two‐way ANOVA with Tukey's multiple comparisons test. ns, not significant; **P < 0.01; **Supplementary Figure S7. TPOR protein expression in Ba/F3 cells**. Western blot analysis of TPOR expression in Ba/F3 cells stably expressing the indicated constructs and sorted for equivalent GFP expression levels; **Supplementary Figure S8. Cell surface expression of TPOR in Ba/F3 cells exposed to TPO‐HT**. Ba/F3 cells expressing TPOR variants were sorted for comparable GFP expression levels and exposed to various TPO‐HT concentrations. Surface expression was assessed by flow cytometry using a PE‐conjugated anti‐HA antibody. Data represent geometric mean ± SD of three independent experiments, except for the S264F 11.0 nM condition, which is based on two independent experiments. Statistical analysis was performed using one‐way ANOVA with Tukey's multiple comparisons test. ns, not significant; *P < 0.05; **Supplementary Figure S9. TPO dose‐response in Ba/F3 cells**. TPO dose‐response in Ba/F3 cells expressing TPOR variants. Cell proliferation was measured after 3 days using a CellTiter‐Glo luminescent cell viability assay in the presence of increasing concentrations of TPO. Data represent mean ± SD of three independent experiments performed in triplicate. Statistical analysis was performed using two‐way ANOVA with Tukey's multiple comparisons test. ns, not significant; *P < 0.05; ****P < 0.0001; **Supplementary Figure S10. Expression of NanoBRET constructs**. Western blot analysis of NanoLuc‐ and HaloTag‐fused TPOR constructs expressed in HEK293T cells; **Supplementary Figure S11. Cell surface expression of HaloTag‐fused TPOR variants**. HEK293T cells transiently expressing HaloTag‐fused TPOR constructs were analyzed by flow cytometry. Cell surface expression levels were assessed in HaloTag ligand‐positive cells using a PE‐conjugated anti‐HA antibody. Data represent geometric mean ± SD of three independent experiments, except for the untransfected control, which is based on two independent experiments. Statistical analysis was performed using one‐way ANOVA with Tukey's multiple comparisons test. ns, not significant; ****P < 0.0001; **Supplementary Figure S12. STAT5 reporter gene assay in HEK293T cells**. JAK‐STAT pathway activation in HEK293T cells transiently transfected with HA‐tagged TPOR variants together with JAK2, STAT5, and a firefly luciferase reporter plasmid (pSPI‐LUC) driven by STAT5‐responsive elements. Cells were stimulated with TPO, Elt, or left unstimulated. Luciferase activity was normalized to Renilla luciferase (pRL‐TK) as an internal control. Data represent mean ± SD of three independent experiments performed in triplicate. Statistical analysis was performed using two‐way ANOVA with Tukey's multiple comparisons test. ns, not significant; ****P < 0.0001; **Supplementary Figure S13. Cell surface expression levels of TPOR variants in HEK293T cells**. HEK293T cells transiently expressing TPOR constructs were analyzed by flow cytometry. Cell surface expression levels were assessed in GFP‐positive cells using a PE‐conjugated anti‐HA antibody. Data represent geometric mean ± SD of three independent experiments. Statistical analysis was performed using one‐way ANOVA with Tukey's multiple comparisons test. ns, not significant; *P < 0.05; ***P < 0.001.

Supporting information

## Data Availability

The data that support the findings of this study are available from the corresponding author upon reasonable request. The author has provided the required Data Availability Statement, and if applicable, included functional and accurate links to said data therein.
