# Supplementary material for: The thrombopoietin receptor S264F mutant drives MPN with marrow fibrosis through a dual mechanism
Source: Hemasphere. 2026 Oct 7;10(10):e70437. doi: 10.1002/hem3.70437 (PMC13644553; doi:10.1002/hem3.70437)
Supplement: Supplementary file 1 [file HEM3-10-e70437-s001.docx]

**Supplementary figure legends**

**Supplementary Figure S1. Peripheral blood counts at diagnosis.**

**Supplementary Figure S2. STAT3 reporter gene assay in HEK293T cells.**

HEK293T cells were transiently transfected with empty vector (EV) or hemagglutinin (HA)-tagged TPOR constructs, together with JAK2 and STAT3, and a STAT3-responsive luciferase reporter (pGL4.47). Cells were stimulated with TPO, eltrombopag (Elt) or left unstimulated. Luciferase activity was normalized to Renilla luciferase (pRL-TK) as an internal control. Data represent mean ± SD of three independent experiments performed in triplicate. Statistical analysis was performed using two-way ANOVA with Tukey’s multiple comparisons test. ns, not significant; **P < 0.01; ****P < 0.0001.

**Supplementary Figure S3. TPOR protein expression in HEK293T cells.**

Western blot analysis of TPOR expression in HEK293T cells transiently transfected with the indicated constructs.

**Supplementary Figure S4. Estimation of TPO-HaloTag concentration.**

Western blot analysis of serial dilutions of TPO-HaloTag (TPO-HT)-containing supernatant (top) alongside a HaloTag standard protein at known concentrations (bottom). Band intensities were compared within the linear detection range of the assay to estimate the concentration of TPO-HT in the original supernatant.

**Supplementary Figure S5. STAT5 reporter gene assay in HEK293T cells.**

JAK-STAT pathway activation in HEK293T cells transiently transfected with HA-tagged TPOR variants together with JAK2, STAT5 and a firefly luciferase reporter plasmid (pSPI-LUC) driven by STAT5-responsive elements. Cells were stimulated with TPO-HT or left unstimulated. Luciferase activity was normalized to Renilla luciferase (pRL-TK) as an internal control. Data represent mean ± SD of three independent transfection experiments. Statistical analysis was performed using two-way ANOVA with Tukey’s multiple comparisons test. ****P < 0.0001.

**Supplementary Figure S6. Flow cytometry-based competitive ligand-binding assay.**

Ba/F3 cells expressing TPOR variants were incubated with Alexa Fluor 660-labeled TPO-HT in the presence or absence of excess untagged TPO. Specific binding was defined as the difference in geometric mean fluorescence intensity between conditions. Data represent geometric mean ± SD of three independent experiments. Statistical analysis was performed using two-way ANOVA with Tukey’s multiple comparisons test. ns, not significant; **P < 0.01.

**Supplementary Figure S7. TPOR protein expression in Ba/F3 cells.**

Western blot analysis of TPOR expression in Ba/F3 cells stably expressing the indicated constructs and sorted for equivalent GFP expression levels.

**Supplementary Figure S8. Cell surface expression of TPOR in Ba/F3 cells exposed to TPO-HT.**

Ba/F3 cells expressing TPOR variants were sorted for comparable GFP expression levels and exposed to various TPO-HT concentrations. Surface expression was assessed by flow cytometry using a PE-conjugated anti-HA antibody. Data represent geometric mean ± SD of three independent experiments, except for the S264F 11.0 nM condition, which is based on two independent experiments. Statistical analysis was performed using one-way ANOVA with Tukey’s multiple comparisons test. ns, not significant; *P < 0.05.

**Supplementary Figure S9. TPO dose-response in Ba/F3 cells.**

TPO dose-response in Ba/F3 cells expressing TPOR variants. Cell proliferation was measured after 3 days using a CellTiter-Glo luminescent cell viability assay in the presence of increasing concentrations of TPO. Data represent mean ± SD of three independent experiments performed in triplicate. Statistical analysis was performed using two-way ANOVA with Tukey’s multiple comparisons test. ns, not significant; *P < 0.05; ****P < 0.0001.

**Supplementary Figure S10. Expression of NanoBRET constructs.**

Western blot analysis of NanoLuc- and HaloTag-fused TPOR constructs expressed in HEK293T cells.

**Supplementary Figure S11. Cell surface expression of HaloTag-fused TPOR variants.**

HEK293T cells transiently expressing HaloTag-fused TPOR constructs were analyzed by flow cytometry. Cell surface expression levels were assessed in HaloTag ligand-positive cells using a PE-conjugated anti-HA antibody. Data represent geometric mean ± SD of three independent experiments, except for the untransfected control, which is based on two independent experiments. Statistical analysis was performed using one-way ANOVA with Tukey’s multiple comparisons test. ns, not significant; ****P < 0.0001.

**Supplementary Figure S12. STAT5 reporter gene assay in HEK293T cells.**

JAK-STAT pathway activation in HEK293T cells transiently transfected with HA-tagged TPOR variants together with JAK2, STAT5 and a firefly luciferase reporter plasmid (pSPI-LUC) driven by STAT5-responsive elements. Cells were stimulated with TPO, Elt or left unstimulated. Luciferase activity was normalized to Renilla luciferase (pRL-TK) as an internal control. Data represent mean ± SD of three independent experiments performed in triplicate. Statistical analysis was performed using two-way ANOVA with Tukey’s multiple comparisons test. ns, not significant; ****P < 0.0001.

**Supplementary Figure S13. Cell surface expression levels of TPOR variants in HEK293T cells.**

HEK293T cells transiently expressing TPOR constructs were analyzed by flow cytometry.

Cell surface expression levels were assessed in GFP-positive cells using a PE-conjugated anti-HA antibody. Data represent geometric mean ± SD of three independent experiments. Statistical analysis was performed using one-way ANOVA with Tukey’s multiple comparisons test. ns, not significant; *P < 0.05; ***P < 0.001.
